# Paeoniflorin mitigates high glucose-induced lifespan reduction by inhibiting insulin signaling in *Caenorhabditis elegans*

**DOI:** 10.3389/fphar.2023.1202379

**Published:** 2023-06-19

**Authors:** Tianwen Liu, Ziheng Zhuang, Dayong Wang

**Affiliations:** ^1^ School of Pharmaceutical Engineering and Life Science, Changzhou University, Changzhou, China; ^2^ Medical School, Southeast University, Nanjing, China

**Keywords:** paeoniflorin, glucose toxicity, *C. elegans*, pharmacological, insulin signaling

## Abstract

In organisms, high glucose can cause several aspects of toxicity, including the lifespan reduction. Paeoniflorin is the major component of Paeoniaceae plants. Nevertheless, the possible effect of paeoniflorin to suppress high glucose toxicity in reducing lifespan and underlying mechanism are largely unclear. Thus, in this study, we examined the possible effect of paeoniflorin in suppressing high glucose (50 mM)-induced lifespan reduction and the underlying mechanism in *Caenorhabditis elegans*. Administration with 16–64 mg/L paeoniflorin could prolong the lifespan in glucose treated nematodes. Accompanied with this beneficial effect, in glucose treated nematodes, expressions of *daf-2* encoding insulin receptor and its downstream kinase genes (*age-1*, *akt-1*, and *akt-2*) were decreased and expression of *daf-16* encoding FOXO transcriptional factor was increased by 16–64 mg/L paeoniflorin administration. Meanwhile, the effect of paeoniflorin in extending lifespan in glucose treated nematodes was enhanced by RNAi of *daf-2*, *age-1*, *akt-1*, and *akt-2* and inhibited by RNAi of *daf-16*. In glucose treated nematodes followed by paeoniflorin administration, the increased lifespan caused by *daf-2* RNAi could be suppressed by RNAi of *daf-16*, suggesting that DAF-2 acted upstream of DAF-16 to regulate pharmacological effect of paeoniflorin. Moreover, in glucose treated nematodes followed by paeoniflorin administration, expression of *sod-3* encoding mitochondrial Mn-SOD was inhibited by *daf-16* RNAi, and the effect of paeoniflorin in extending lifespan in glucose treated nematodes could be suppressed by *sod-3* RNAi. Molecular docking analysis indicated the binding potential of paeoniflorin with DAF-2, AGE-1, AKT-1, and AKT-2. Therefore, our results demonstrated the beneficial effect of paeoniflorin administration in inhibiting glucose-induced lifespan reduction by suppressing signaling cascade of DAF-2-AGE-1-AKT-1/2-DAF-16-SOD-3 in insulin signaling pathway.

## Introduction

Hyperglycemia is a pathological situation with severe increase in blood glucose level in the patients (Gunst et al., 2019). This increase in blood glucose level is the major cause for type 2 diabetes (Monnier et al., 2012), and may be induced by sedentary lifestyle and excessive calories intake (Stumvoll et al., 2005; Taylor et al., 2021). Hyperglycemia is also closely associated with some other diseases, such as diabetic cardiomyopathy and renal disease (Baldwin and Apel, 2013; Jia et al., 2018). Hyperglycemia is further related to increased mortality and morbidity during neonatal period (Ramel and Rao, 2020). Chronic hyperglycemia has emerged as a global health challenge. Considering the biological activity and non-toxicity, the efforts to identify beneficial natural extracts or compounds have been conducted in order to be used for treating hyperglycemia.

Various plants and their metabolic products have been focused as an important area for identifying bioactive compounds with the health benefits, and most of these bioactive compounds are secondary metabolites and present at relatively small amount in plants (Aiello et al., 2019; Sahardi and Makpol, 2019). As a classic model animal with well-described genetic backgrounds, both molecular and metabolic pathways in *Caenorhabditis elegans* are highly conserved and have corresponding homologues in humans (Pir et al., 2017; Watts and Ristow, 2017; Wang, 2019). *C. elegans* has been shown as a powerful animal model for evaluating different aspects pharmacological effects of bioactive compounds, including anti-bacterial and fungal infections and anti-neurodegeneration diseases (such as ant-Alzheimer’s disease) (Parker et al., 2004; Griffin et al., 2017; Madende et al., 2020). During the past decade, many efforts have been made to discover bioactive compounds having anticipated pharmacological effects, such as anti-aging using the *C. elegans* as aging model (Collins et al., 2006; Okoro et al., 2021). In addition, due to sensitivity to environmental exposure and small volume of exposure (Wang, 2020; Xu et al., 2022a; Xu et al., 2022b; Zhao Y.-L. et al., 2023; Hua et al., 2023c), *C. elegans* is useful for high throughput screen of drugs or compounds (Braungart et al., 2004; O’Reilly et al., 2014).

*C. elegans* has been frequently applied to assess glucose-induced toxicity and to identify compounds against glucose toxicity (Fitzenberger et al., 2013; Yan et al., 2017a). In *C. elegans*, some components in biochemical reactions were found to have potential in reducing the glucose toxicity. For example, supplementation with carnitine (a substrate of β-oxidation) or diosgenin (a phytosterol substitute) could reduce glucose toxicity via nuclear hormone receptor DAF-12 or insulin signaling (Deusing et al., 2015a; Shanmugam et al., 2017). Besides these, some plant extracts or bioactive compounds were also found to have the potential against glucose-induced toxicity. Green tea extract enriched with catechin or blackberry leaf extract prevented the glucose-induced survival reduction (Fitzenberger et al., 2014; Deusing et al., 2015b). The anthocyanin in mulberry fruit and C-glycosides in *Apios americana* leaves also exhibited the protective function against the toxicity under hyperglycemic condition (Yan et al., 2017a; Yan et al., 2017b).

Paeoniflorin is the major component of total glycoside paeony. Initially, the monoterpenoid glycoside paeoniflorin was extracted in Paeonia lactiflora Pall (Zhang et al., 2019). The content of paeoniflorin in different species of Paeoniaceae ranges from 0.05% to 10.7% (Zhang X. et al., 2022). Some reports have suggested the function of paeoniflorin treatment in inhibiting glucose-induced inflammation response and oxidative injury (Shao et al., 2016; Yang et al., 2016; Zhu et al., 2017). In this study, we employed *C. elegans* as an animal model to further determine the possible beneficial effect of paeoniflorin against glucose toxicity in reducing lifespan and the underlying mechanism. In *C. elegans*, insulin signaling pathway plays a crucial function in regulating longevity (Kenyon, 2010). The insulin receptor DAF-2 regulates the longevity by activating downstream kinase cascade (AGE-1-AKT-1/2), and inhibiting FOXO transcription factor DAF-16/FOXO (Lin et al., 2001; Zheng et al., 2018). Our results suggest that administration with paeoniflorin could inhibit the glucose toxicity in reducing lifespan by suppressing insulin signaling in nematodes. Our data suggested the potential effect of paeoniflorin treatment in suppressing glucose-induced lifespan reduction.

## Materials and methods

### Reagent

**FIGURE 1:**
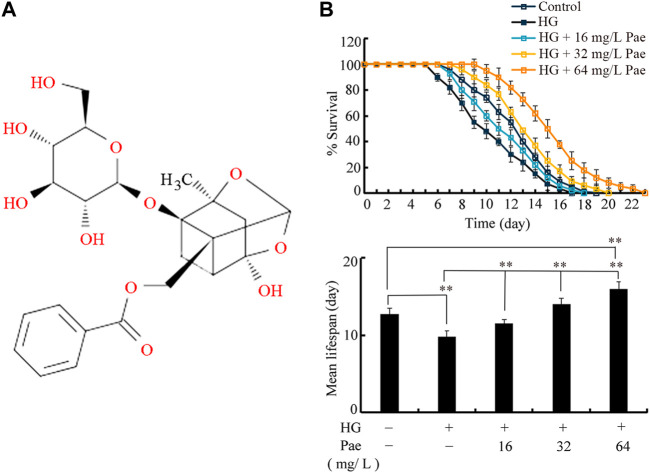
Effect of paeoniflorin administration in reducing glucose toxicity. **(A)** Chemical structure of paeoniflorin. **(B)** Effect of paeoniflorin administration on lifespan in glucose treated nematodes. Lifespan curve of HG was significantly (*p* < 0.01) different from control. Lifespan curves of HG + 16 mg/L Pae, HG + 32 mg/L Pae, and HG+64 mg/L Pae were significantly (*p* < 0.01) different from HG. Lifespan curve of HG + 64 mg/L Pae was significantly (*p* < 0.01) different from control. HG, high glucose (50 mM); Pae, paeoniflorin. **p < 0.01.

The paeoniflorin was purchased from Yuanye Bio-Technology Co., Ltd., (Shanghai, China). The purity of paeoniflorin was ≥98%. Chemical structure of paeoniflorin is shown in Figure 1A. The stocking solution (1.024 mg/mL) was prepared by dissolving paeoniflorin into DMSO. The working solutions of paeoniflorin were prepared by diluting the stocking solution with K buffer and stored at 4°C. The control solution used in this experiment was comprised of DMSO, and diluted with K buffer in the same way as paeoniflorin solutions.

### *C. elegans* maintenance

The used *C. elegans* strains are listed in Supplementary Table S1. *C. elegans* are normally maintained on nematode growth medium (NGM) agar plates seeded with *E. coli* OP50 as food source according to the standard protocol (Brenner, 1974). Both *C. elegans* and *E. coli* strains were obtained from *Caenorhabditis* Genetics Center (CGC).

### Preparation of glucose toxicity model in *C. elegans*

To assess the glucose toxicity, L1-larvae were treated in NGM plates fed with OP50 and containing glucose. The final concentration of added _D_-glucose was 50 mM in NGM plates (Shanmugam et al., 2017). To obtain synchronized L1-larval nematodes, eggs were released from pregnant hermaphrodites by treating them with solution lysis buffer (0.45 M NaOH and 2% HOCl), and allowed to further develop into L1-larvae (Zhang et al., 2022b). The nematodes were exposed to 50 mM glucose till to L4-larvae (approximately 2.5-day).

### Pharmacological treatment

After the exposure of nematodes to 50 mM glucose, the nematodes were transferred into paeoniflorin solutions with the addition of OP50 to further treat for 48 h. The examined concentrations for paeoniflorin were 25, 50, and 100 mg/L, which were basically selected as previously described (Hua et al., 2023a). After the paeoniflorin treatment, the examined nematodes were transferred onto normal NGM plates for lifespan analysis. The experiments were repeated three times.

### Lifespan analysis

The lifespan was analyzed as described (Zhang et al., 2022a). After glucose and paeoniflorin treatments, the survival of nematodes was counted on normal NGM plates. The survival was checked every day. The animals were counted as dead if no response of pharynx was observed after prodding with a platinum wire. To exclude the effect from offspring, we transferred the nematodes daily to new NGM plates. During the lifespan assay, median lifespan refers to the day at which 50% nematodes survive. For each treatment, 50 nematodes were tested. Three replicates were performed. Significance between lifespan curves was analyzed by Kaplan-Meier software, followed by the log-rank test.

### Transcriptional expression analysis

Total RNA of nematodes was extracted using TRIZOL, and then reverse transcribed to obtain cDNA. Quality of prepared RNAs was assessed in NanoDrop One based on the ratio of OD260/OD280. The quantitative real-time polymerase chain reaction (qRT-PCR) was performed using SYBR Green Master Mix in an ABI 7500 real-time PCR system. The method of comparative CT (ΔΔ CT) was employed to evaluate transcriptional expressions of genes in *C. elegans*. The expressions of examined genes were expressed after normalization to reference gene *tba-1* encoding tubulin (Hua et al., 2022). Three replicates were carried out. The designed primers for reference and examined genes are shown in Supplementary Table S2.

### RNA interference (RNAi)

The *E. coli* HT115 expressing dsRNA for certain gene was used to feeding the examined nematodes (Zhao Y. et al., 2022). The RNAi feeding was carried out at the stage of pharmacological treatment with paeoniflorin. The HT115 RNAi clones for *daf-2*, *age-1*, *akt-1*, *akt-2*, *daf-16*, and *sod-3* were obtained from Source Bioscience (Cambridge, United Kingdom) (Rual et al., 2004). HT115 expressing L4440, an empty vector, was employed as a control (Liu et al., 2022). RNAi efficiency was assessed based on qRT-PCR analysis, which is shown in Supplementary Figure S1.

### Molecular docking analysis

The molecular interaction between paeoniflorin and proteins was performed using computer stimulation analysis. The structure of paeoniflorin was downloaded from the PubChem database (https://pubchem.ncbi.nlm.nih.gov/), and the structure of proteins of DAF-2, AGE-1, AKT-1, and AKT-2 were obtained from the UniProt database (https://www.rcsb.org). The Openbel software is used to convert the structures to PDB format (https://openbabel.org). Using AutoDock software (https://vina.scripps.edu/), the binding of paeoniflorin with certain proteins was stimulated, and different docking poses with the best affinity were generated. Using PyMol software (https://www.pymol.org/), image optimization and generation were performed.

### Safety evaluation of paeoniflorin administration

We used lifespan, locomotion behavior, pumping rate, and brood size as endpoints to perform the safety evaluation of paeoniflorin administration. Under the normal condition, the L4-larval nematodes were treated with 16–64 mg/L paeoniflorin for 48 h. The lifespan was analyzed as described above. Locomotion behaviors of head thrash and body bend were analyzed to reflect alteration in function of motor neurons (Wang et al., 2023b; Hua et al., 2023d). A head thrash is defined as one swing of nematode body, and a body bend refers to the crawling of one wavelength (Zhao Y.-Y. et al., 2023). Fifty nematodes were assayed for each treatment. Pumping rate was used to evaluate the pharyngeal pumping, and analyzed as described (Wu et al., 2016). Nematodes were first normally maintained on NGM plate for 1-h. After that, pumping rate was assessed in 1 min intervals. Fifty nematodes were assayed for each treatment. Reproductive capacity was reflected by the endpoint of brood size (Zhao Y.-Y. et al., 2022; Hua et al., 2023e). Brood size was considered as number of offspring until nematodes end up laying eggs (Hua et al., 2023b). Thirty nematodes were assayed for each treatment.

### Statistical analysis

Data are presented as means ± standard derivation (SD). SPSS 12.0 software was used for statistical analysis. Differences between different groups were analyzed by analysis of variance (ANOVA). Using SPSS Statistics 25.0 software, the significances of differences between exposure groups were analyzed using one-way or two-way analysis of variance (ANOVA) followed by Least-Significant Difference (LSD) of the *post hoc* test. Two-way ANOVA was used for comparing multiple factors. A probability level of 0.01 was considered statistically significant.

## Results

### Paeoniflorin treatment increased lifespan in nematodes after glucose exposure

In *C. elegans*, treatment with 50 mM glucose significantly reduced the lifespan as indicated by both lifespan curves and mean lifespan (Figure 1B). Under the background of 50 mM glucose treatment, administration with 16–64 mg/L paeoniflorin could obviously increase the lifespan of nematodes (Figure 1B). In 50 mM glucose treated nematodes, administration with 64 mg/L paeoniflorin even induced higher lifespan than control group (Figure 1B).

### Paeoniflorin administration altered expressions of *daf-2*, *age-1*, *akt-1*, *akt-2*, and *daf-16* in glucose treated nematodes

**FIGURE 2:**
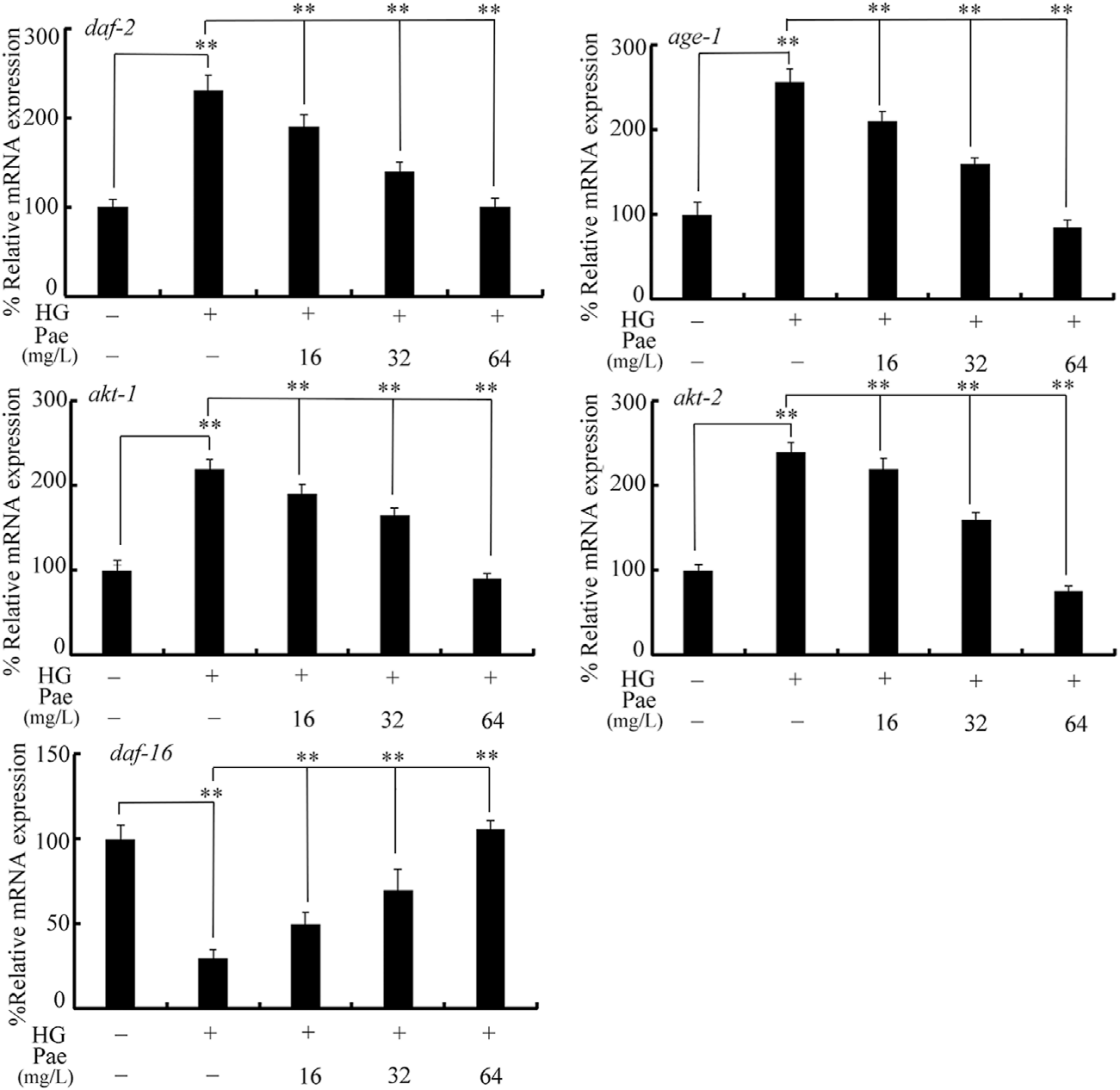
Effect of paeoniflorin administration on expressions of *daf-2*, *age-1*, *akt-1*, *akt-2*, and *daf-16* in 50 mM glucose treated nematodes. HG, high glucose (50 mM); Pae, paeoniflorin. **p < 0.01.

Considering the important function of insulin signaling pathway during aging control (Lin et al., 2001), we examined the effect of paeoniflorin administration on expressions of *daf-2*, *age-1*, *akt-1*, *akt-2*, and *daf-16* in 50 mM glucose treated nematodes. Treatment with glucose (50 mM) significantly increased expressions of *daf-2*, *age-1*, *akt-1*, and *akt-2*, and decreased *daf-16* expression (Figure 2). In 50 mM glucose treated nematodes, the decrease in *daf-2*, *age-1*, *akt-1*, and *akt-2* expression and the increase in *daf-16* expression could be significantly reversed by following administration with paeoniflorin (16–64 mg/L) to different degrees (Figure 2).

### Paeoniflorin administration affected expression of DAF-16::GFP in glucose treated nematodes

**FIGURE 3:**
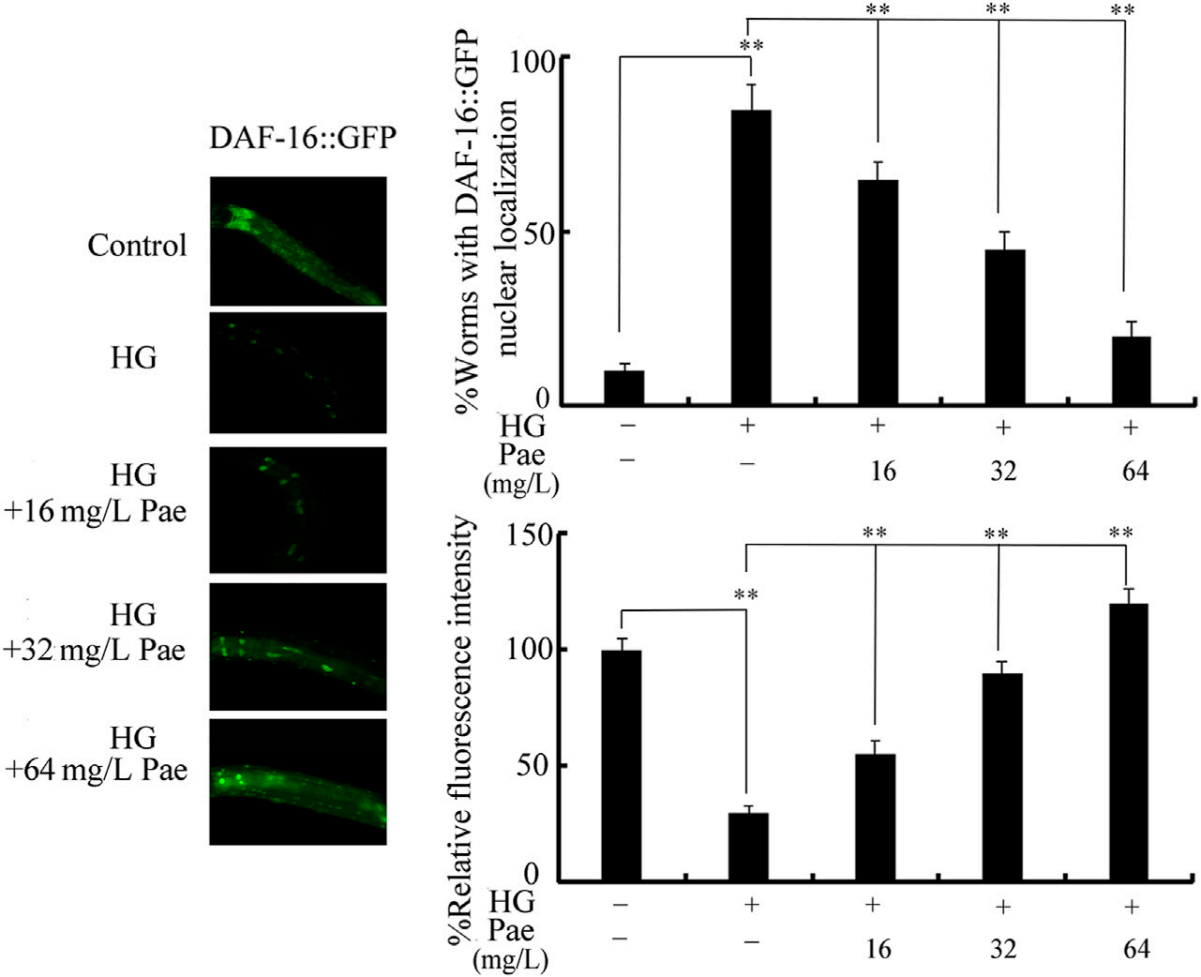
Effect of paeoniflorin administration on expression of DAF-16::GFP in 50 mM glucose treated nematodes. For each treatment, 50 nematodes were tested. HG, high glucose (50 mM); Pae, paeoniflorin. **p < 0.01.

We further used transgenic strain of TJ356 to investigate effect of paeoniflorin administration on DAF-16::GFP expression in 50 mM glucose treated nematodes. Treatment with 50 mM glucose caused increase in DAF-16::GFP translocation in the nucleus and decrease in relative fluorescence intensity of DAF-16::GFP (Figure 3). In 50 mM glucose treated nematodes, administration with 16–64 mg/L paeoniflorin could cause the change of DAF-16::GFP translocation from nucleus to cytoplasm and increase in relative fluorescence intensity of DAF-16::GFP (Figure 3).

### RNAi of *daf-2*, *age-1*, *akt-1*, *akt-2*, and *daf-16* altered the pharmacological effect of paeoniflorin in increasing lifespan in glucose treated nematodes

**FIGURE 4:**
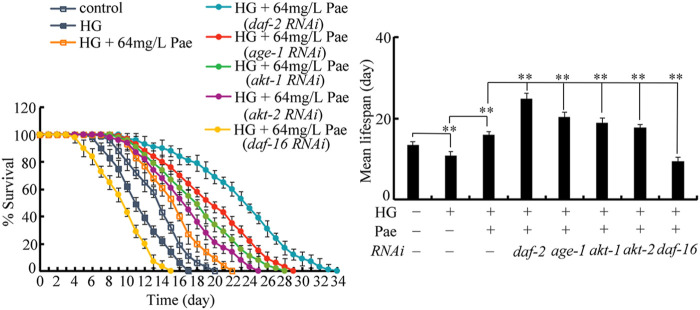
Effect of *daf-2*, *age-1*, *akt-1*, *akt-2*, and *daf-16* RNAi on pharmacological effect of paeoniflorin (64 mg/L) in increasing lifespan in 50 mM glucose treated nematodes. Lifespan curve of HG was significantly (*p* < 0.01) different from control. Lifespan curve of HG + 64 mg/L Pae was significantly (*p* < 0.01) different from HG. Lifespan curves of HG + Pae + *daf-2* (*RNAi*), HG + Pae + *age-1* (*RNAi*), HG + Pae + *akt-1* (*RNAi*), HG + Pae + *akt-2* (*RNAi*), and HG + Pae + *daf-16* (*RNAi*) were significantly (*p* < 0.01) different from HG + Pae. HG, high glucose (50 mM); Pae, paeoniflorin. **p < 0.01.

To determine the exact function of insulin signaling pathway, we investigated the effect of *daf-2*, *age-1*, *akt-1*, *akt-2*, and *daf-16* RNAi on the role of paeoniflorin (64 mg/L) in increasing lifespan in 50 mM glucose treated nematodes. After glucose treatment followed by paeoniflorin administration, the lifespan was significantly increased by RNAi of *daf-2*, *age-1*, *akt-1*, and *akt-2* compared to wild-type, and meanwhile the lifespan was significantly decreased by *daf-16* RNAi compared to wild-type (Figure 4). Therefore, the insulin signaling pathway was involved in controlling pharmacological effect of paeoniflorin in increasing lifespan in glucose treated nematodes.

### Genetic interaction between DAF-2 and DAF-16 in regulating pharmacological effect of paeoniflorin in increasing lifespan in glucose treated nematodes

**FIGURE 5:**
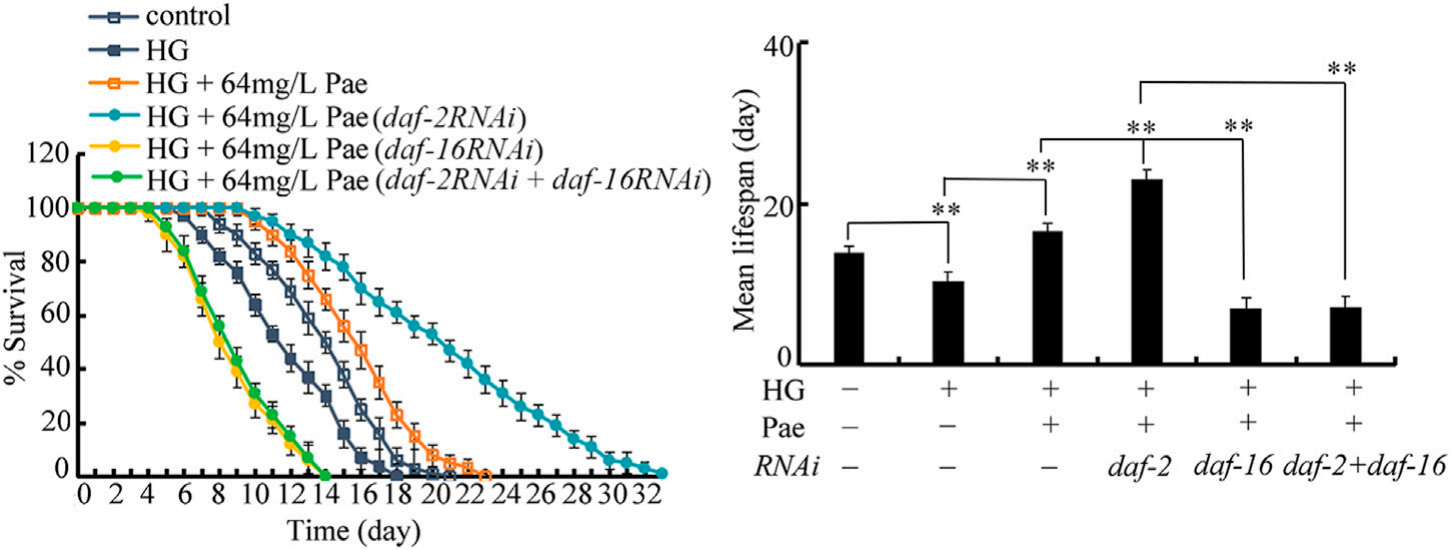
Genetic interaction between *daf-2* and *daf-16* in regulating function of paeoniflorin (64 mg/L) in increasing lifespan in 50 mM glucose treated nematodes. Lifespan curve of HG was significantly (*p* < 0.01) different from control. Lifespan curve of HG + 64 mg/L Pae was significantly (*p* < 0.01) different from HG. Lifespan curves of HG + Pae + *daf-2* (*RNAi*) and HG + Pae + *daf-16* (*RNAi*) were significantly (*p* < 0.01) different from HG + Pae. Lifespan curve of HG + Pae + *daf-16* (*RNAi*); *daf-2* (*RNAi*) was significantly (*p* < 0.01) different from HG + Pae + *daf-2*(*RNAi*). HG, high glucose (50 mM); Pae, paeoniflorin. **p < 0.01.

To determine the genetic interaction between DAF-2 and DAF-16 in regulating the pharmacological effect of paeoniflorin, double RNAi of *daf-2* and *daf-16* was performed after the 50 mM glucose treatment. In glucose treated nematodes followed by paeoniflorin administration, the lifespan of *daf-16(RNAi);daf-2(RNAi)* nematodes was similar to that of *daf-16(RNAi)* nematodes (Figure 5), which suggested that DAF-16 acted downstream of DAF-2 to regulate the pharmacological effect of paeoniflorin in increasing lifespan in glucose treated nematodes.

### SOD-3 acts as downstream target of DAF-16 to regulate pharmacological effect of paeoniflorin in increasing lifespan in glucose treated nematodes

**FIGURE 6:**
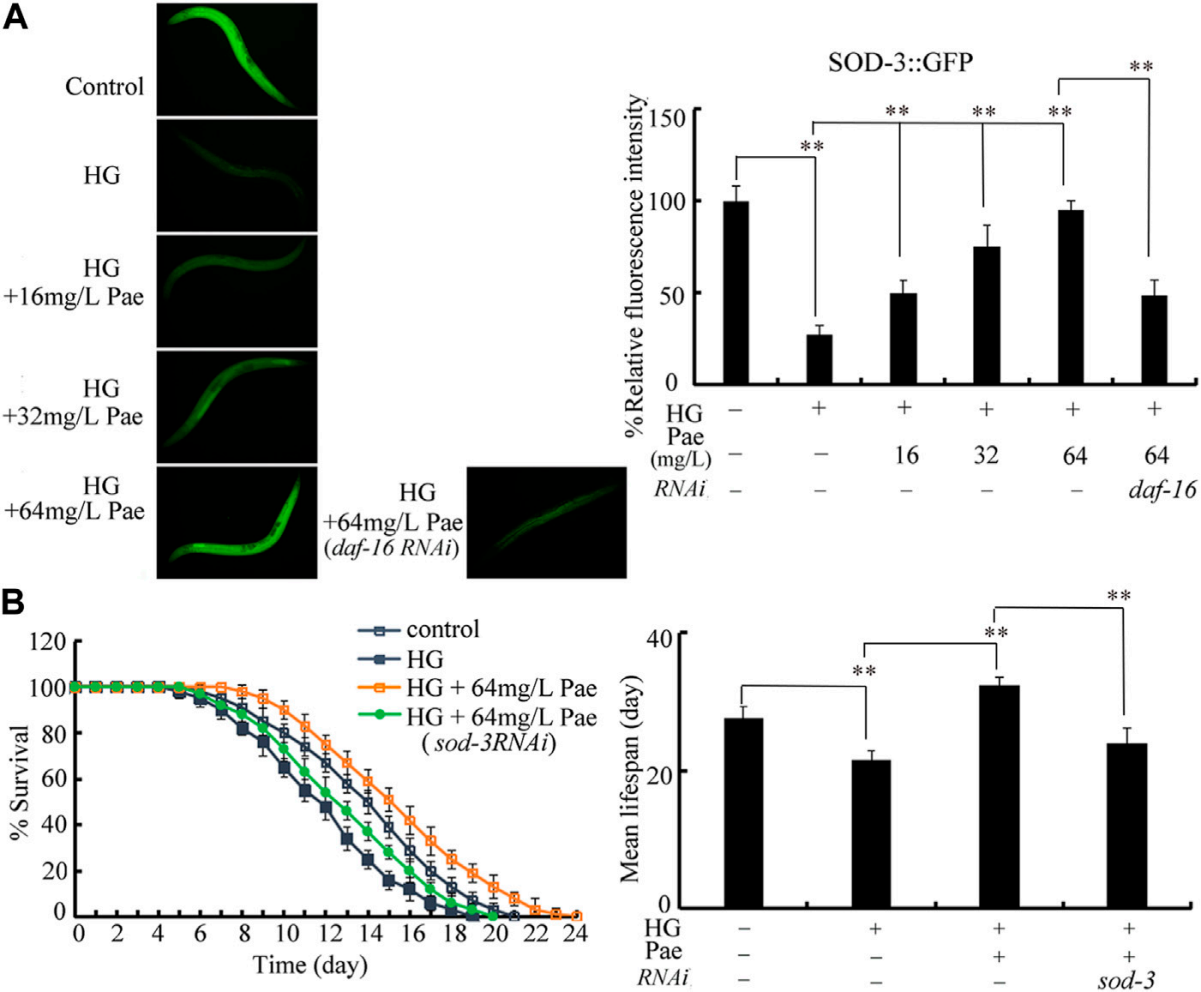
Role of SOD-3 in regulating function of paeoniflorin (64 mg/L) in increasing lifespan in 50 mM glucose treated nematodes. **(A)** Effect of *daf-16* RNAi on SOD-3::GFP expression in 50 mM glucose treated nematodes after paeoniflorin (64 mg/L) administration. **(B)** Effect of *sod-3* RNAi on lifespan in 50 mM glucose treated nematodes after paeoniflorin (64 mg/L) administration. HG, high glucose (50 mM); Pae, paeoniflorin. **p < 0.01.

During the control of stress response, SOD-3 is a primary target of DAF-16 (Shao et al., 2019; Wang, 2019). Using transgenic strain CF1553, the decrease in SOD-3::GFP expression caused by 50 mM glucose could be suppressed by administration with 16–64 mg/L paeoniflorin (Figure 6A). The function of paeoniflorin (64 mg/L) in increasing SOD-3::GFP expression in 50 mM glucose treated nematodes was inhibited by RNAi of *daf-16* (Figure 6A). Meanwhile, we observed that the role of paeoniflorin (64 mg/L) in increasing lifespan in 50 mM glucose treated nematodes was suppressed by RNAi of *sod-3* (Figure 6B).

### Binding potential between paeoniflorin and DAF-2, AGE-1, AKT-1, and AKT-2

**FIGURE 7:**
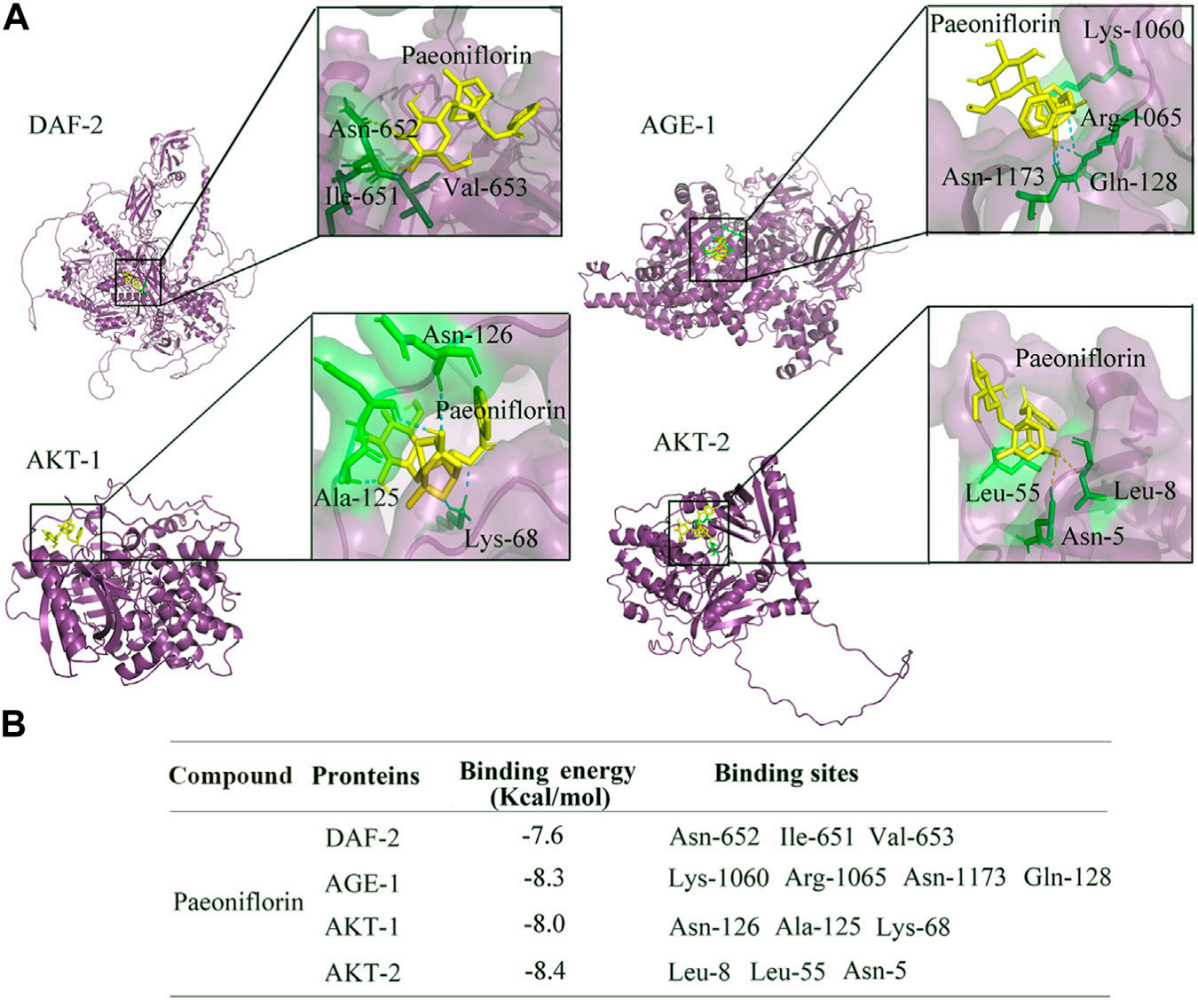
Binding potential of paeoniflorin with DAF-2, AGE-1, AKT-1, and AKT-2. **(A)** Molecular docking between paeoniflorin and DAF-2, AGE-1, AKT-1, or AKT-2. **(B)** The binding energy of paeoniflorin bound to the active sites of DAF-2, AGE-1, AKT-1, or AKT-2.

To further confirm the relationships between paeoniflorin and DAF-2 and its downstream kinases, molecular docking method was used to investigate the specific binding sites of paeoniflorin in DAF-2, AGE-1, AKT-1, and AKT-2. The molecular docking analysis showed that paeoniflorin potentially interacts with the amino acid residues of asparagine (Asn)-652, isoleucine (Ile)-651, and valine (Val)-653 in DAF-2, the amino acid residues of lysine (Lys)-1060, arginine (Arg)-1065, asparagine (Asn)-1173, and glutamine (Gln)-128 in AGE-1, amino acid residues of asparagine (Asn)-126, alanine (Ala)-125, and lysine (Lys)-68 in AKT-1, and amino acid residues of asparagine (Asn)-5 and leucine (Leu)-8 and (Leu)-55 in AKT-2 via hydrogen bonding (Figure 7A). The docked stable confirmations showed the binding energies between paeoniflorin and DAF-2, AGE-1, AKT-1, and AKT-2 were −7.6, −8.3, −8, and −8.4 kcal/mol, respectively (Figure 7B). These results suggested the binding potentials of paeoniflorin to DAF-2 and its downstream kinases.

### Safety evaluation of paeoniflorin administration on nematodes

**FIGURE 8:**
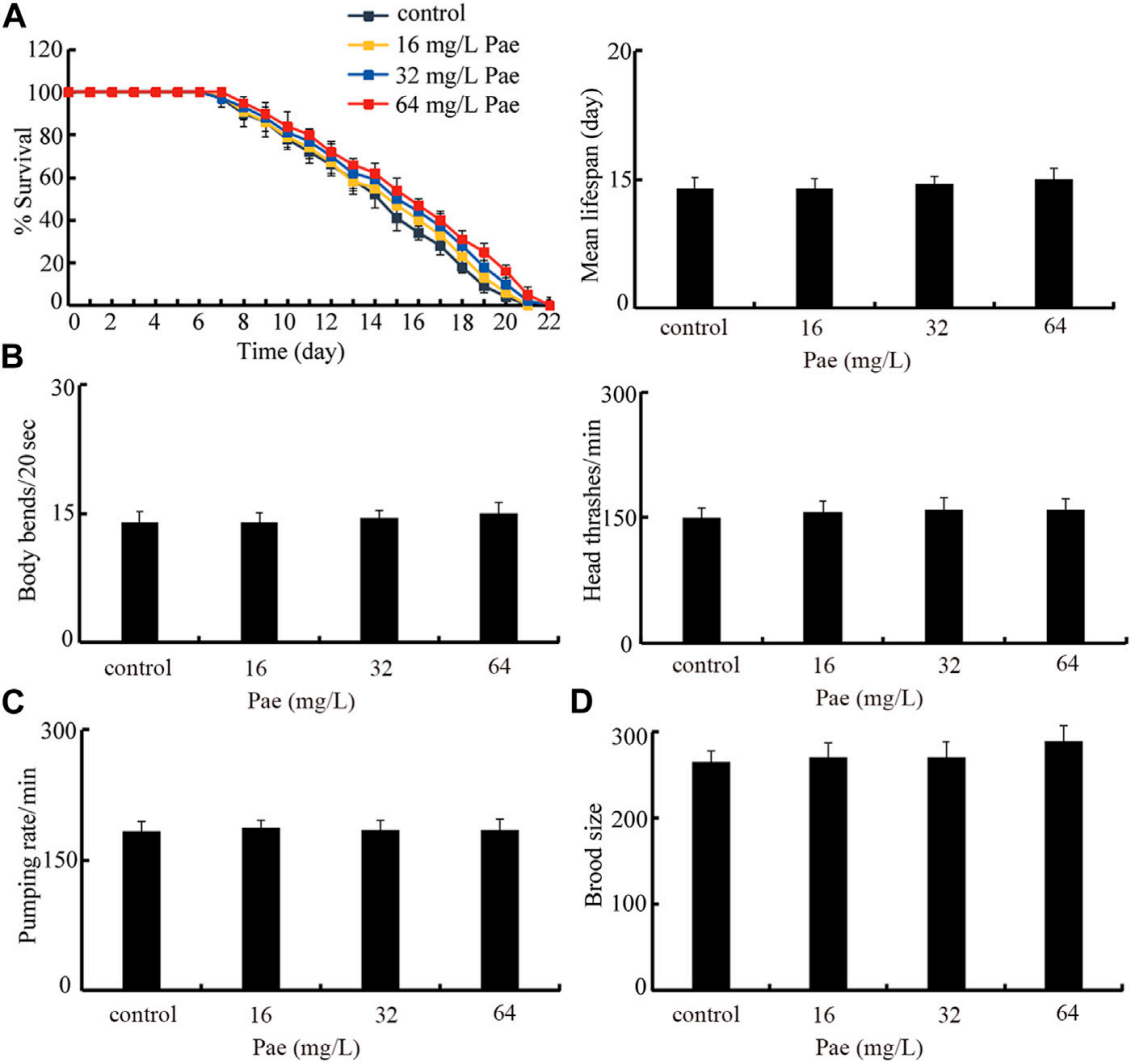
Safety assessment of 16–64 mg/L paeoniflorin on lifespan **(A)**, locomotion behavior **(B)**, pumping rate **(C)**, and brood size **(D)**. Pae, paeoniflorin.

Finally, we selected lifespan, locomotion behavior, pumping rate, and brood size as endpoints to evaluate the possible safety of paeoniflorin administration at these aspects in nematodes. Under the normal condition, administration with 16–64 mg/L paeoniflorin did not obviously affect lifespan, locomotion behavior reflected by body bend and head thrash, pumping rate, and brood size (Figures 8A–D).

## Discussion

The paeoniflorin has multiple aspects of pharmacological effects, including anti-inflammation, neuroprotective effect, and anticancer (Xiang et al., 2020; Zhou et al., 2020; Hong et al., 2022). In *C. elegans*, paeoniflorin was first observed to have function in inhibiting age-onset Aβ proteotoxicity by partially regulating oxidative stress response (Ai et al., 2018). Recently, it was found that paeoniflorin treatment could attenuate polystyrene nanoparticle-induced damage on reproductive capacity and germline development (Hua et al., 2023a). In addition, paeoniflorin treatment was helpful for nematodes against *Pseudomonas aeruginosa* infection and biofilm formation (Wang et al., 2023a). In this study, we further observed that treatment with 16–64 mg/L paeoniflorin could inhibit the glucose toxicity in reducing lifespan in nematodes (Figure 1). Previous observations on the pharmacological effect of paeoniflorin treatment against glucose-induced oxidative injury partially support our finding (Yang et al., 2016). Our data suggested the novel pharmacological effect of paeoniflorin treatment against glucose toxicity.

In *C. elegans*, although several signaling pathways have been identified to be required for control of longevity (Lapierre and Hansen, 2012; Martins et al., 2016; Blackwell et al., 2019), the insulin signaling pathway plays the central role and is an evolutionarily conserved mechanism (Barbieri et al., 2003; Murphy and Hu, 2013). In this insulin signaling pathway, the activated insulin receptor DAF-2 suppresses the longevity by increasing downstream several kinases (AGE-1, AKT-1, and AKT-2) and inhibiting FOXO transcriptional factor DAF-16 (Braeckman and Vanfleteren, 2007). In glucose treated nematodes, we observed the increase in *daf-2*, *age-1*, *akt-1*, and *akt-2* expressions and decrease in *daf-16* expression (Figure 2), which suggested that the observed lifespan reduction by glucose treatment was associated with the activation of insulin signaling. Moreover, in the glucose treated nematodes, 16–64 mg/L paeoniflorin administration could further obviously decrease *daf-2*, *age-1*, *akt-1*, and *akt-2* expressions and increase *daf-16* expression (Figure 2). Meanwhile, Moreover, in the glucose treated nematodes, we also observed the increase in DAF-16::GFP expression after paeoniflorin administration (Figure 3). That is, the observed beneficial effect of paeoniflorin against glucose toxicity on lifespan was due to the suppression in signaling cascade of DAF-2-AGE-1-AKT-1/2 and the activation of DAF-16 in nematodes.

In glucose treated nematodes, the obvious increase in translocation of DAF-16::GFP into nucleus was observed (Figure 3). The increase in translocation of DAF-16::GFP into nucleus has been also detected in nematodes exposed to other stresses (such as simulated microgravity) or pollutants (such as graphene oxide) (Zhao et al., 2016; Kong et al., 2019), suggesting that the translocation of DAF-16::GFP into nucleus is a normally formed response to stresses or pollutants. That is, the stresses and toxicants with certain degree of toxicity can induce both the decrease in fluorescence intensity of DAF-16::GFP and the translocation of DAF-16::GFP into nucleus. Moreover, the following administration with 16–64 mg/L paeoniflorin could suppress this translocation of DAF-16::GFP into nucleus in glucose treated nematodes (Figure 3). This implied the important function of paeoniflorin against toxicity of stresses, including the glucose treatment.

The functional analysis demonstrated that the beneficial effect of paeoniflorin against glucose toxicity in reducing lifespan could be enhanced by RNAi of *daf-2*, *age-1*, *akt-1*, and *akt-2*, and inhibited by RNAi of *daf-16* (Figure 4). These observations confirmed the functions of DAF-2, AGE-1, AKT-1/2, and DAF-16 in regulating the pharmacological effect of paeoniflorin in inhibiting glucose toxicity. The insulin signaling was also required for controlling the pharmacological effects of compounds or plant extracts in extending lifespan under normal condition (Wang et al., 2018; Zeng et al., 2021; Zhou et al., 2021). For example, the DAF-16/DAF-2 insulin signaling regulated the pharmacological effect of sulforaphane in promoting lifespan and healthspan (Qi et al., 2021). That is, under both the normal and stress conditions, the insulin signaling is required for the control of pharmacological effect of compounds in extending longevity in nematodes.

The genetic interaction analysis indicated that, in glucose treated nematodes, DAF-2 acted upstream of DAF-16 to regulate the pharmacological effect of paeoniflorin in extending lifespan (Figure 5). Similarly, DAF-2 could function upstream of DAF-16 to control pharmacological effect of luteolin to promote bacterial pathogen resistance (Xiao et al., 2023). DAF-2 could also act upstream of DAF-16 to regulate toxicity on lifespan induced by nanoplastic particle and simulated microgravity (Kong et al., 2019; Shao et al., 2019; Liu et al., 2022). Therefore, the signaling cascade of DAF-2-DAF-16 is a conserved mechanism for the regulation of both pharmacological effects of compounds and stress response in nematodes.

During the control of pharmacological effect of paeoniflorin, SOD-3/Mn-SOD was identified as the downstream target of DAF-16. Two lines of evidence were raised in this study. Firstly, the increase in SOD-3::GFP expression induced by 16–64 mg/L paeoniflorin in glucose treated nematodes could be suppressed by RNAi of *daf-16* (Figure 6A). Secondly, the pharmacological effect of paeoniflorin in extending lifespan in glucose treated nematodes could be inhibited by RNAi of *sod-3* (Figure 6B). Therefore, the DAF-16 could target to SOD-3 to affect the pharmacological effect of paeoniflorin on lifespan in glucose treated nematodes. SOD-3 was also identified to act as downstream target of DAF-16 during the control of pharmacological effect of sulforaphane and blueberry extract in extending the lifespan (Wang et al., 2018; Qi et al., 2021).

In this study, the molecular docking analysis indicated the binding potentials of paeoniflorin to DAF-2, AGE-1, AKT-1, and AKT-2 (Figure 7). That is, the administrated paeoniflorin can bind to both insulin receptor DAF-2 and its downstream three kinases, which provides an important signal amplification mechanism for paeoniflorin to exert its pharmacological effect in nematodes. Moreover, this further supported the molecular mechanism that paeoniflorin could extend lifespan in glucose treated nematodes by inhibiting the signaling cascade of DAF-2-AGE-1-AKT-1/2, which further activated the DAF-16 and its target of SOD-3.

Finally, with the aid of several endpoints including lifespan, locomotion behavior, pumping rate, and brood size, our results suggested the safety of paeoniflorin administration in nematodes (Figure 8). The safe property of paeoniflorin administration has also been shown in other reports (Li et al., 2016; Ngo et al., 2019). Paeoniflorin is one of the main bioactive ingredients in the blood for Xuebijing injection with the safety to be used to treat the sepsis in the clinical (Li et al., 2018; Xiao et al., 2018), which indirectly supports the safety of paeoniflorin administration.

## Conclusion

Together, using the high-glucose model in *C. elegans*, we examined the potential of paeoniflorin administration against glucose toxicity in reducing lifespan. Our results demonstrated the potential of 16–64 mg/L paeoniflorin against glucose toxicity in reducing lifespan. This beneficial effect of paeoniflorin administration was associated with the inhibition of insulin signaling pathway reflected by the decrease in *daf-2*, *age-1*, *akt-1*, and *akt-2* expressions and the increase in *daf-16* expression. During the control of pharmacological effect of paeoniflorin, DAF-2 acted upstream of DAF-16. After pharmacological treatment, the DAF-16 further activated its target of SOD-3, suggesting the requirement of DAF-2-AGE-1-AKT-1/2-DAF-16-SOD-3 signaling cascade for formation of pharmacological effect of paeoniflorin in inhibiting glucose toxicity. In this signaling cascade, molecular docking analysis indicates the binding potential of paeoniflorin to DAF-2, AGE-1, AKT-1, and AKT-2. Our data highlights the potential of paeoniflorin administration in inhibiting toxicity of high glucose on longevity of organisms.

## Data Availability

The datasets presented in this study can be found in online repositories. The names of the repository/repositories and accession number(s) can be found in the article/Supplementary Material.
