# Supplementary material for: Paeoniflorin mitigates high glucose-induced lifespan reduction by inhibiting insulin signaling in Caenorhabditis elegans
Source: Front Pharmacol. 2023 Jun 19;14:1202379. doi: 10.3389/fphar.2023.1202379 (PMC10315627; doi:10.3389/fphar.2023.1202379)
Supplement: Supplementary file 1 [file DataSheet1.doc]

**Supporting Information:**


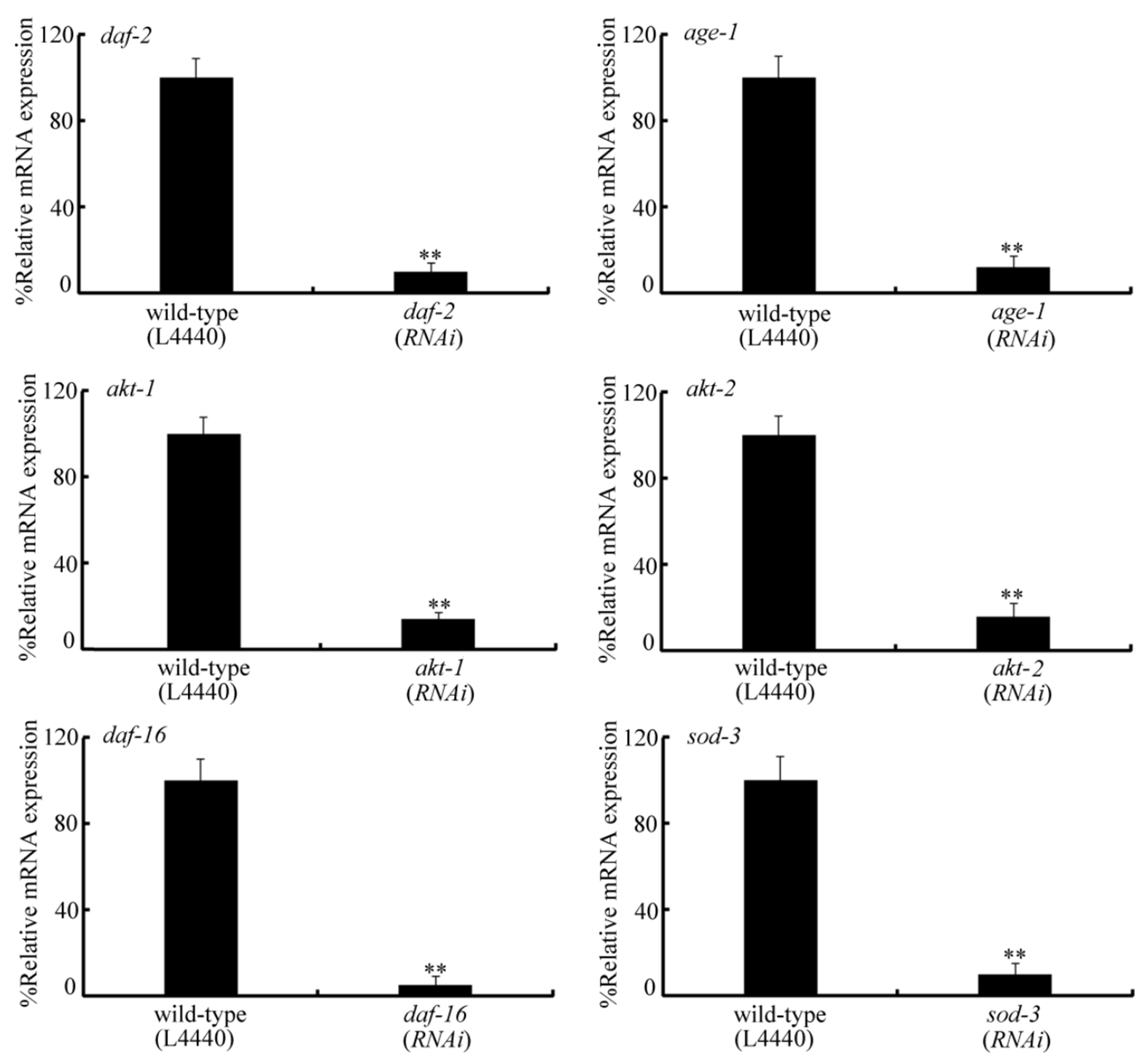


**Fig. S1** RNAi efficiency of *daf-2*, *age-1*, *akt-1*, *akt-2*, and *sod-3*. ^**^*P* < 0.01 vs wild-type(L4440).

**Table S1.** Information for *C. elegans* strains

| Strains | Genotype | Description |
| --- | --- | --- |
| N2 |  | Wild-type |
| CF1553 | *muIs84*[(pAD76) sod-3p::GFP + rol-6(su1006)] | Transgenic strain expressing SOD-3::GFP |
| TJ356 | *zIs356*[daf-16p::daf-16a/b::GFP + rol-6(su1006)] | Transgenic strain expressing DAF-16::GFP |

**Table S2.** Primer information for qRT-PCR

| Gene | Forward primer (5’-3’) | Reverse primer (5’-3’) |
| --- | --- | --- |
| *daf-2* | GCTTACGCGATGAGCTGTGAT | TCGCTGGCGACTATGTGA |
| *age-1* | GGAAAGACCAAACTTGGGATC | AGGCTTCGACGCATAACG |
| *akt-1* | GACGAACTTCTGCCGACT | GGACAACCGTTTCCTGAG |
| *akt-2* | AAGGTTCCTTGACCGAGA | ATCAGCCGTTACCAGAGC |
| *daf-16* | ACATTGCTCGAAGTGCCGAA | CATTGCTGTCGACCCGTTTG |
| *tba-1* | TCAACACTGCCATCGCCGCC | TCCAAGCGAGACCAGGCTTCAG |
